# Autonomic Control of Heart Rate During Sleep Is Depressed in Young Children With Prader–Willi Syndrome

**DOI:** 10.1111/jsr.70094

**Published:** 2025-05-09

**Authors:** Okkes R. Patoglu, Lisa M. Walter, Georgina Plunkett, Margot J. Davey, Gillian M. Nixon, Bradley A. Edwards, Rosemary S. C. Horne

**Affiliations:** ^1^ Department of Paediatrics Monash University Melbourne Australia; ^2^ Melbourne Children's Sleep Centre Monash Children's Hospital Melbourne Australia; ^3^ Department of Physiology Biomedicine Discovery Institute and School of Psychological Sciences, Monash University Melbourne Australia

**Keywords:** heart rate variability, nocturnal dipping, paediatric, Prader–Willi syndrome, sleep

## Abstract

Children with Prader–Willi syndrome are at increased risk of both obstructive and central sleep apnoea. In addition, these children have impaired autonomic control, which may be exacerbated by sleep apnoea. The aim of this study was to compare autonomic control using heart rate variability and nocturnal dipping of heart rate in children with Prader–Willi syndrome and typically developing children. We identified 50 children with Prader–Willi syndrome and matched them for age, obstructive and central apnoea‐hypoponea index, body mass index and sex to 50 typically developing children. All children underwent overnight polysomnography. Time and frequency domain heart rate variability were analysed during N2, N3, REM and total sleep, and nocturnal dipping of heart rate from wake was calculated. Children with Prader–Willi syndrome had reduced time domain heart rate variability in REM, reduced low frequency power in N2, higher heart rate in REM and total sleep (*p* < 0.05 for all) and reduced fall in heart rate from wake to REM (*p* < 0.05). When stratified into age groups, similar results were found in children ≤ 1 and > 1 ≤ 6 years, with no differences between groups in children > 6 years of age. The significant reduction in LF power and nocturnal dipping indicates children with Prader–Willi syndrome have delayed maturation of autonomic control, particularly below 6 years of age. Investigating the impact of age on heart rate variability longitudinally and treatments such as growth hormone remains to be elucidated.

## 1 Introduction

Prader–Willi syndrome (PWS) is a complex congenital disorder with variable manifestations including physical, physiological and psychological abnormalities (Gillett and Perez 2016) that affects between 1/10,000 and 25,000 live births (Cassidy et al. 2012). Adults with PWS have an increased risk of cardiovascular morbidity and mortality (Butler et al. 2017) and have a significantly higher relative risk of adverse cardiovascular outcomes when compared to the general population (Hedgeman et al. 2017). Life expectancy in people with PWS is significantly reduced to approximately 30 years, with cardiac causes being the second most common cause of death (Butler et al. 2017), making a better understanding of cardiovascular physiology important in this group. While the increased risk has been attributed to abnormal electrocardiogram (ECG) findings (Marcus et al. 2012a) and dysfunction of autonomic cardiovascular control (DiMario et al. 1994; Butler et al. 2023), the precise mechanisms responsible remain elusive. In addition, children with PWS are at substantially higher risk of obstructive sleep apnoea (OSA) (Schluter et al. 1997; Tan and Urquhart 2017), with incidences as high as 57% (Abel et al. 2019) compared to 1%–6% (Marcus et al. 2012b) in typically developing (TD) children, and central sleep apnoea (CSA) with incidences of 25%–72% (Lu et al. 2020) compared to 4%–6% in TD children (Kritzinger et al. 2011; Felix et al. 2016). Both OSA and CSA have been shown to negatively affect cardiovascular control in TD children (O'Driscoll et al. 2011; Nisbet et al. 2014); however, previous studies that investigated cardiovascular control in children with PWS have not controlled for OSA or CSA (Brito et al. 2021; Richer et al. 2023; Debs et al. 2024).

Cardiovascular control can be assessed using time domain and frequency domain heart rate variability (HRV) which provide information on both the sympathetic and parasympathetic arms of the autonomic nervous system (ANS). There have only been three previous studies assessing HRV in children with PWS, which found conflicting results (Brito et al. 2021; Richer et al. 2023; Debs et al. 2024). All three studies did not control for either OSA or CSA, and also included some children on growth hormone (GH) treatment (Brito et al. 2021; Richer et al. 2023) or all children were on GH (Debs et al. 2024), which has been found to impact the ANS (Leong et al. 2000, 2001). One study found no difference in either time or frequency domain HRV during wakefulness (Richer et al. 2023), whereas the two other studies found reduced total HRV and parasympathetic modulation during sleep (Brito et al. 2021; Debs et al. 2024) when compared to TD children.

Heart rate (HR) dipping during sleep, a phenomenon that is thought to be restorative to the cardiovascular system, is a risk factor for cardiovascular mortality and is associated with poorer cardiovascular outcomes, (Ben‐Dov et al. 2007) has also been used to assess autonomic control in TD children (Francis et al. 2024); however, there have been no studies in children with PWS.

Given the limited and conflicting literature on autonomic control in children with PWS, the aim of this study was to compare autonomic cardiovascular control in GH naïve children with PWS to that of TD children, controlling for age, OSA and CSA, body mass index (BMI) and sex. We hypothesised that compared to TD children, children with PWS would have reduced autonomic cardiovascular control.

## 2 Methods

Ethical approval for this study was granted by Monash Health (RES22‐0000035 L) and Monash University Human Research Ethics Committees. This was a retrospective observational study where children with PWS (aged < 18 years) referred for polysomnography (PSG) at the Melbourne Children's Sleep Centre between December 2010 (when electronic sleep study records were available) and December 2023 were eligible if their parents had given consent at the time of the PSG for their data to be utilised for research purposes. Sleep studies were either performed for assessment of breathing during sleep as required by local guidelines prior to initiation of GH treatment, or for suspected OSA or CSA based on symptoms. If multiple PSGs were performed prior to GH initiation, then the last study closest to GH initiation was selected. Each child with PWS was matched to a TD child who had been referred for assessment of sleep disordered breathing (SDB) based on age (± 1 year, or ± 6 months in infants ≤ 1 year of age), BMI z‐score in children > 2 years of age (in same category, with ≥ 1.04 as overweight and ≥ 1.65 as obese), and where possible, sex and BMI in children < 2 (Shypailo 2020). OSA severity was matched by severity category (obstructive apnoea‐hypopnoea index (OAHI) ≤ 1 events/h as primary snoring, > 1 < 5 events/h as mild, ≥ 5 < 10 events/h as moderate and ≥ 10 events/h as severe OSA) and ± 2 events/h. CSA severity was matched for severity category (central apnoea‐hypopnoea index (CAHI) ≤ 5 events/h as normal and > 5 events/h as CSA) and ± 2 events/h.

Overnight attended PSG studies were conducted by experienced paediatric sleep scientists in compliance with guidelines established by the American Academy of Sleep Medicine (AASM) for paediatric sleep studies at the time of the study (Iber et al. 2007; Berry et al. 2012, 2017, 2020), using a commercially available PSG system (E‐Series or Grael, Compumedics, Melbourne, Australia). The setup included electroencephalogram, electrooculogram, submental electromyogram, tibialis muscle electromyogram, electrocardiogram (ECG) (sampled at rate of 512 Hz), abdominal and thoracic respiratory plethysmography, oronasal thermistor, nasal pressure cannula, transcutaneous carbon dioxide (TcCO_2_) and peripheral oxygen saturation (SpO_2_) (Bitmos, Düsseldorf, Germany or Masimo, Irvine, CA) set to a 2‐s averaging time.

Studies were manually staged and scored using Compumedics ProFusion software. For studies recorded prior to 2012, NREM 3 and NREM 4 were combined into N3 to be consistent with current staging rules. Total sleep time (TST) was defined as the total time spent asleep, sleep latency as the time taken to reach the first epoch of sleep, sleep efficiency as the percentage of TST of the time available for sleep. Wake after sleep onset (WASO) was defined as the time spent awake during the sleep period and the arousal index as the total number of arousal events per hour of sleep. The OAHI was defined as the total number of obstructive and mixed apnoeas and hypopnoeas per hour of TST and was used to define OSA severity. In addition, the CAHI was defined as the total number of central apnoeas and hypopnoeas per hour of TST and was used to define the presence of CSA. The respiratory disturbance index (RDI) was defined as the total number of obstructive, central and mixed apnoeas and hypopnoeas per hour of sleep. SpO_2_ nadir was the lowest oxygen saturation point during TST and the average SpO_2_ drop was the average drop in SpO_2_ that occurred following a respiratory event.

### 2.1 Heart Rate Variability (HRV) Analysis

Sleep study data including the ECG were transferred via European Data Format (EDF) for HRV analysis. HRV quantifies the alterations in the ANS by measuring the oscillations in the intervals between consecutive heart beats (R‐R intervals) (Malik et al. 1996). Time domain and spectral analysis were performed on spontaneous changes in R‐R interval in 2 min bins using peak detection and the Lomb‐Scargle Periodogram (LabChart 8.1.21, ADInstruments, Sydney, Australia). This technique is based upon the same fundamental theory as the Fast Fourier transformation but is superior as it does not require an evenly sampled data set—it allows for the inherent variability of the R‐R interval data and hence the tachogram can be transformed directly without an intervening approximation stage. From the entire overnight study, including respiratory events, all 2‐min bins of the same sleep stage and free of movement artefact (ECG disruption by gross body movement) were selected. Each 2‐min bin was separated from the previous bin by at least one 30 s ECG artefact free epoch (Walter et al. 2013). Data from the 2‐min bins from each sleep stage (N2, N3 and REM) were subsequently grouped by sleep stage and a mean value of HRV parameters for each sleep stage was calculated for each subject. To be included in the analysis, each patient required ≥ 6 min of HRV data (≥ 3 bins of data) in each individual sleep stage and at least 4 h of sleep while not on any form of respiratory support.

The power spectral densities for the LF band (0.04–0.15 Hz) reflect a combination of parasympathetic and sympathetic activity, and the HF band (0.15–0.40 Hz) reflects parasympathetic activity (Malik et al. 1996). Total Power reflects the overall autonomic activity, and the power spectral densities are presented as absolute power in ms^2^. The LF/HF ratio was determined as a measure of sympathovagal balance. Time‐domain parameters calculated included SDRR (the standard deviation of R‐R intervals) which reflects contributions from both parasympathetic and sympathetic activity and is highly correlated with LF and Total Power; RMSSD (the square root of the mean of the sum of the squares of differences between adjacent RR intervals) reflects parasympathetic activity; and pRR50 (the percentage of successive RR intervals differing by > 50 ms) also reflects parasympathetic activity. From birth to middle and late childhood, there is significant development of autonomic control—with the majority of development occurring in the first 6 months of life (Harteveld et al. 2021). In this growth period, parasympathetic activity increases and sympathetic activity decreases with postnatal age (Yiallourou et al. 2013). To account for the known effects of age, we grouped the children into three age groups: children ≤ 1 year, > 1 ≤ 6 years and > 6 years of age. In addition, as the HF band is a reflection of respiratory activity, the HF band was defined as the frequency band between the 10th and 90th centiles of the respiratory frequency in children ≤ 6 months of age (Andriessen et al. 2003).

### 2.2 Nocturnal Dipping Analysis

Nocturnal dipping of HR was calculated as the percentage change in HR from quiet wake (before sleep onset) to the first period of N2, N3 and REM sleep and combined for total sleep. The first sleep cycle was chosen for the analysis to target the point of maximal HR dipping, as minimum HR levels have been previously reported in the first 1–2 h of sleep in adults (Snyder et al. 1964), which corresponds to the first sleep cycle. The first sleep cycle started with the first cluster of three or more contiguous epochs of N2, N3, or REM sleep after sleep onset and continued until interrupted by 3 or more contiguous epochs of wake or another sleep stage (Francis et al. 2024). At least 2 artefact‐free minutes of each sleep stage and wake before sleep onset were required for a child to be included in this analysis. In each child, the mean HR for each period analysed was expressed as the percentage change from that child's mean HR during wake before sleep onset. A sleep HR to wake HR ratio was also quantified using the average HR value for quiet wake before sleep onset and total sleep. Based on their sleep‐to‐wake HR ratio, each child was classified according to their level of nocturnal HR dipping as: extreme dipper (ratio ≤ 0.8); dipper (0.8 < ratio ≤ 0.9); non‐dipper (< 0.9 ratio ≤ 1.0); reverse dipper (ratio > 1.0) (Fagard 2009).

### 2.3 Statistical Analysis

Statistical analysis was carried out via SPSS (Version 29.0. Armonk, NY: IBM Corp). Data were first assessed for normality and equal variance. Sleep and respiratory characteristics were compared via an independent sample Student's *t*‐test for parametric data or the Mann–Whitney U test for non‐parametric data. As HR matures with age, and both obstructive and central events can impact autonomic control, frequency and time domain HRV, as well as nocturnal dipping measurements, were compared via linear mixed‐effects modelling covarying for age, OAHI and CAHI for N2, N3, REM and for total sleep. Nocturnal dipping profiles and proportions of children with OSA and CSA were analysed via the Chi‐square test. As 52% (26/50) of children with PWS in our cohort were < 2 years of age, and only 12/26 (46%) of these children < 2 years had height recorded, we did not covary for BMI z‐score due to the high number of missing data points. However, we matched our children individually as closely as possible for age and BMI. Data are presented as median and interquartile range and a *p* value of < 0.05 was considered statistically significant.

## 3 Results

Of the 51 children with PWS who had a PSG between December 2010 and December 2023 and gave consent for the use of data in research, 1 child was excluded as they spent the entire night on respiratory support during their PSG, leaving 50 children who met the inclusion criteria for the study. HRV data during N3 were excluded for one child who had less than 6 min of artefact‐free N3 data. Three infants with PWS whose sleep was staged as quiet and active sleep were only included in the analysis for REM and total sleep and not N2 or N3 sleep. HRV data for the period of the PSG when children with PWS were on respiratory support (9 on supplemental O_2_ and 1 on continuous positive airway pressure) was removed from the analysis (*n* = 10), while their sleep characteristics were calculated over the entire night; their respiratory characteristics were calculated only using the diagnostic portion of the PSG when they were not on respiratory support.

Demographic, sleep and respiratory characteristics are compared in Table 1. By design, there were no differences between groups for age, BMI z‐score, OAHI or CAHI. The children with PWS had greater sleep efficiency and a lower arousal index compared to the TD children, but no other differences in sleep architecture. The average SpO_2_ drop with respiratory events was greater, and the SpO_2_ nadir was lower in the children with PWS compared to the TD children.

**TABLE 1:** Demographic, sleep and respiratory characteristics of children with Prader–Willi syndrome (PWS) and typically developing (TD) children. Values are median (IQR) or count (frequency).

	PWS	TD	*p*
*N*	50	50	
Sex	22F (44%)	22F (44%)	
Age (months)	23.0 (10.0, 71.5)	23.0 (10.0, 65.5)	*p* = 0.812
BMI z‐score*	1.5 (0.9, 2.3)	1.3 (0.5, 1.9)	*p* = 0.162
Total sleep time (min)	458.5 (430.4, 478.1)	438.0 (388.8, 476.0)	*p* = 0.092
Sleep latency (min)	15.5 (5.5, 31.3)	18.3 (8.4, 31.0)	*p* = 0.285
Wake after sleep onset (%)	10.0 (6.7, 14.0)	13.0 (7.0, 18.2)	*p* = 0.123
Sleep efficiency (%)	86.3 (82.1, 90.2)	82.7 (76.1, 87.0)	***p* = 0.019**
NREM (%)	74.5 (69.1, 78.4)	75.4 (70.5, 80.0)	*p* = 0.298
N1 (%)	7.5 (5.4, 10.5)	7.7 (5.2, 11.2)	*p* = 0.521
N2 (%)	43.2 (36.7, 51.5)	39.6 (35.5, 47.2)	*p* = 0.336
N3 (%)	23.4 (16.5, 27.0)	25.8 (21.0, 29.7)	*p* = 0.071
REM (%)	25.3 (21.2, 30.4)	24.7 (19.0, 29.5)	*p* = 0.328
RDI (events/h)	4.8 (2.6, 8.5)	4.4 (2.3, 8.4)	*p* = 0.632
OAHI (events/h)	0.1 (0.0, 0.8)	0.1 (0.0, 0.8)	*p* = 0.371
OAHI ≤ 1 (events/h)	42 (84%)	40 (80%)	*p* = 0.870
OAHI > 1 < 5 (events/h)	5 (10%)	7 (14%)	*p* = 0.585
OAHI ≥ 5 < 10 (events/h)	2 (4%)	2 (4%)	*p* = 1.000
OAHI > 10 (events/h)	1 (2%)	1 (2%)	*P* = 1.000
CAHI (events/h)	3.9 (1.5, 7.7)	2.8 (1.6, 6.3)	*p* = 0.717
CAHI ≤ 5 (events/h)	33 (66%)	34 (68%)	*p* = 0.925
CAHI > 5 (events/h)	17 (34%)	16 (32%)	*p* = 0.880
Arousal index (events/h)	8.6 (6.3, 11.8)	14.1 (11.8, 19.2)	***p* < 0.001**
SpO_2_ nadir (%)	84.0 (74.0, 88.3)	90.0 (85.8, 92.0)	***p* < 0.001**
Average SpO_2_ drop (%)	5.0 (4.0, 6.0)	4.0 (3.0, 5.0)	***p* < 0.001**

*Note*: *BMI z‐score data is only available for *n* = 21 children with PWS and *n* = 24 controls. Bold indicates significant value.

Abbreviations: CAHI, central apnoea‐hypopnoea index; NREM, non‐rapid eye movement; OAHI, obstructive apnoea‐hypopnoea index; RDI, respiratory disturbance index; REM, rapid eye movement.

**FIGURE 1:**
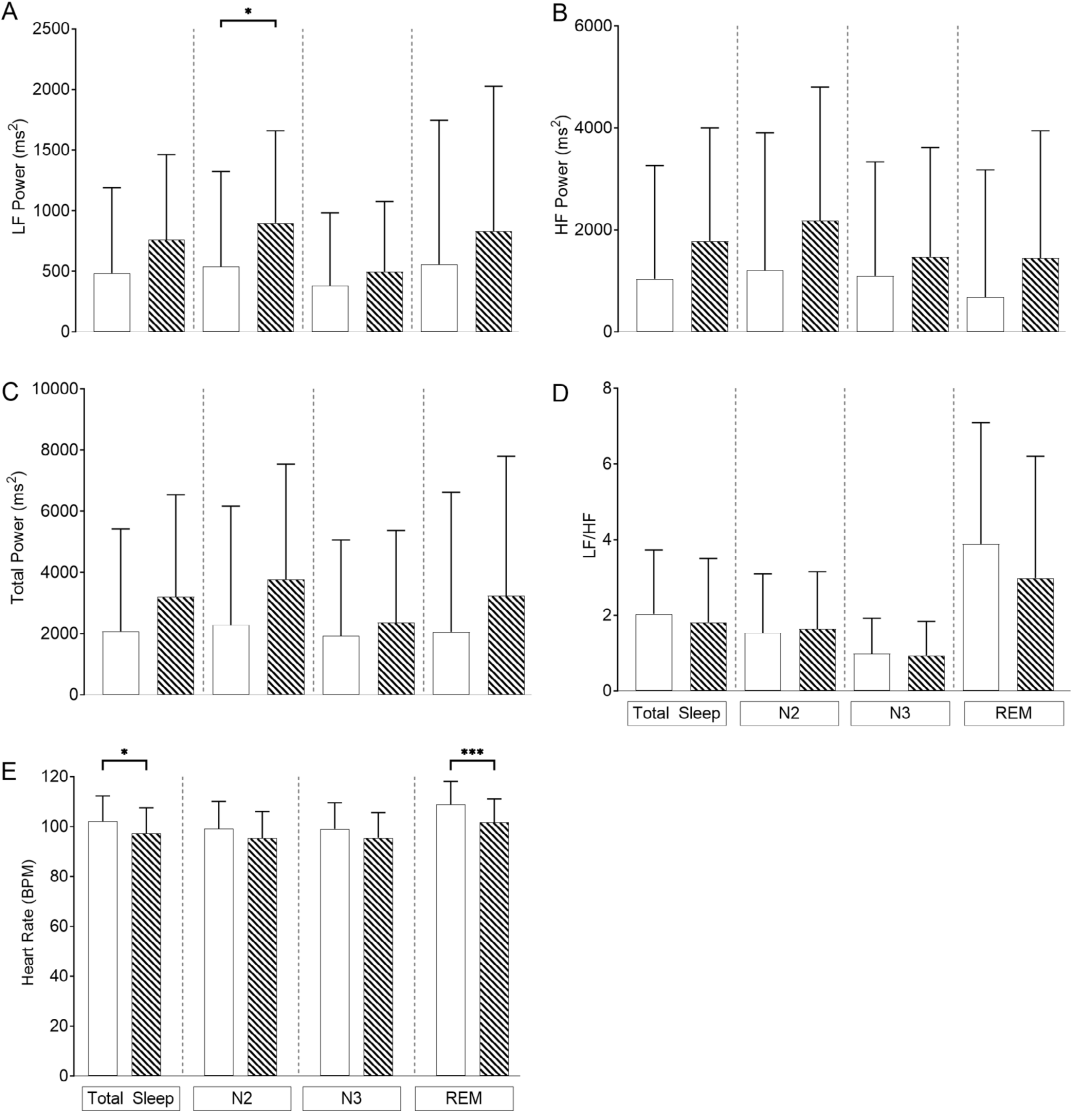
Differences in heart rate and frequency domain heart rate variability parameters using linear mixed‐effects modelling in all children covarying for age (months), obstructive apnoea‐hypopnoea index (OAHI) and central apnoea‐hypopnoea index (CAHI) in children with Prader‐Willi syndrome (white bars) (*n* = 50) versus typically developing (TD) children (hashed bars) (*n* = 50) for (A) LF power; (B) HF power; (C) total power; (D) sympathovagal balance (LF/HF) and (E) heart rate. Values are the adjusted mean and standard deviation. *p < 0.05, ***p < 0.001.

When mixed‐model analysis was conducted adjusting for age, OAHI and CAHI (Figure 1), children with PWS had significantly lower LF in N2, and HR was significantly higher during total sleep and REM sleep compared to TD children.

**FIGURE 2:**
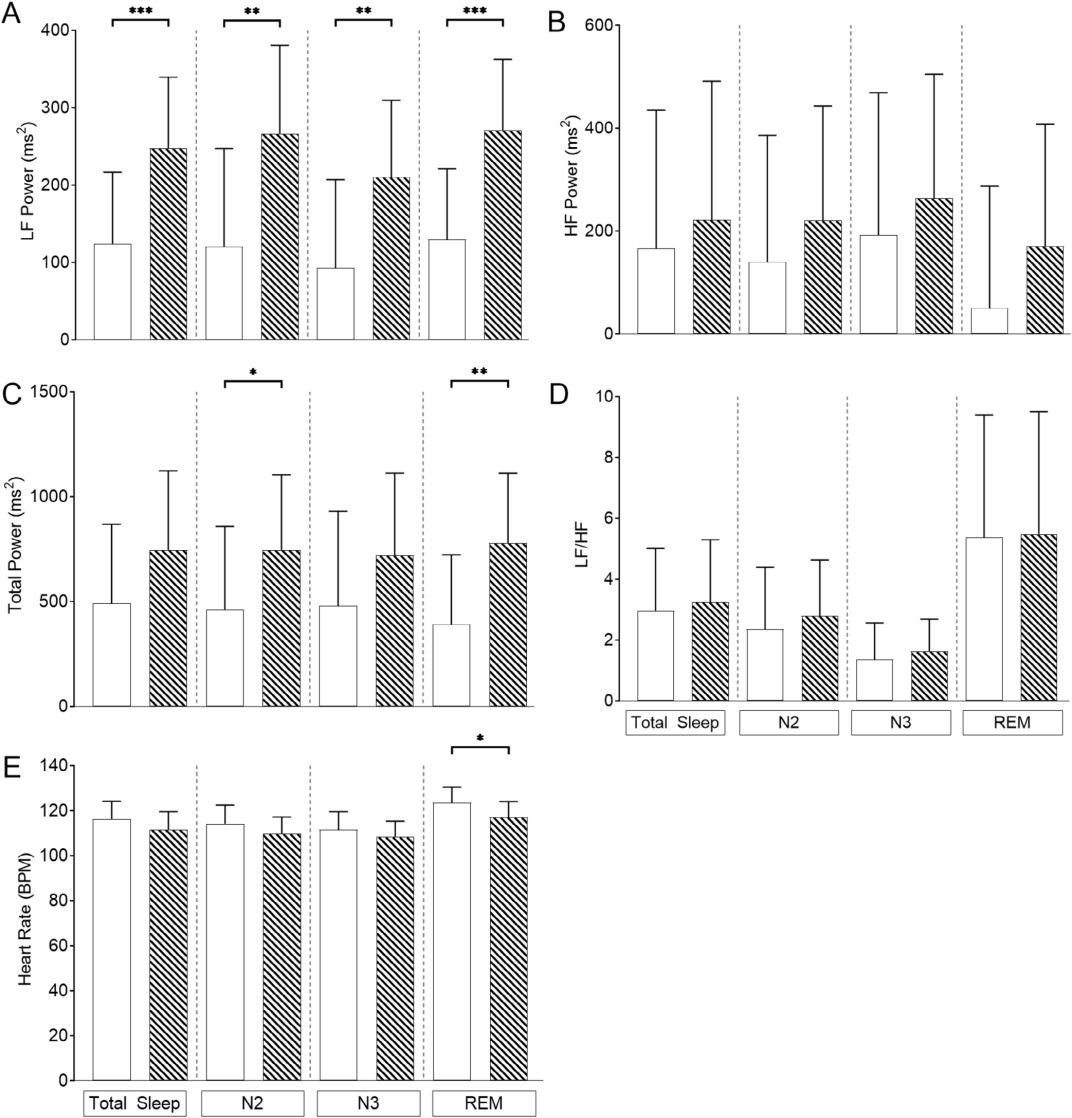
Differences in heart rate and frequency domain heart rate variability parameters using linear mixed‐effects modelling in children ≤ 1‐year covarying for age, obstructive apnoea‐hypopnoea index (OAHI) and central apnoea‐hypopnoea index (CAHI) in children with Prader‐Willi syndrome (white bars) (*n* = 17) and typically developing (TD) children (hashed bars) (*n* = 17) for (A) LF power; (B) HF power; (C) total power; (D) sympathovagal balance (LF/HF) and (E) heart rate. Values are the adjusted mean and standard deviation. *p < 0.05, **p < 0.01, ***p < 0.001.

**FIGURE 3:**
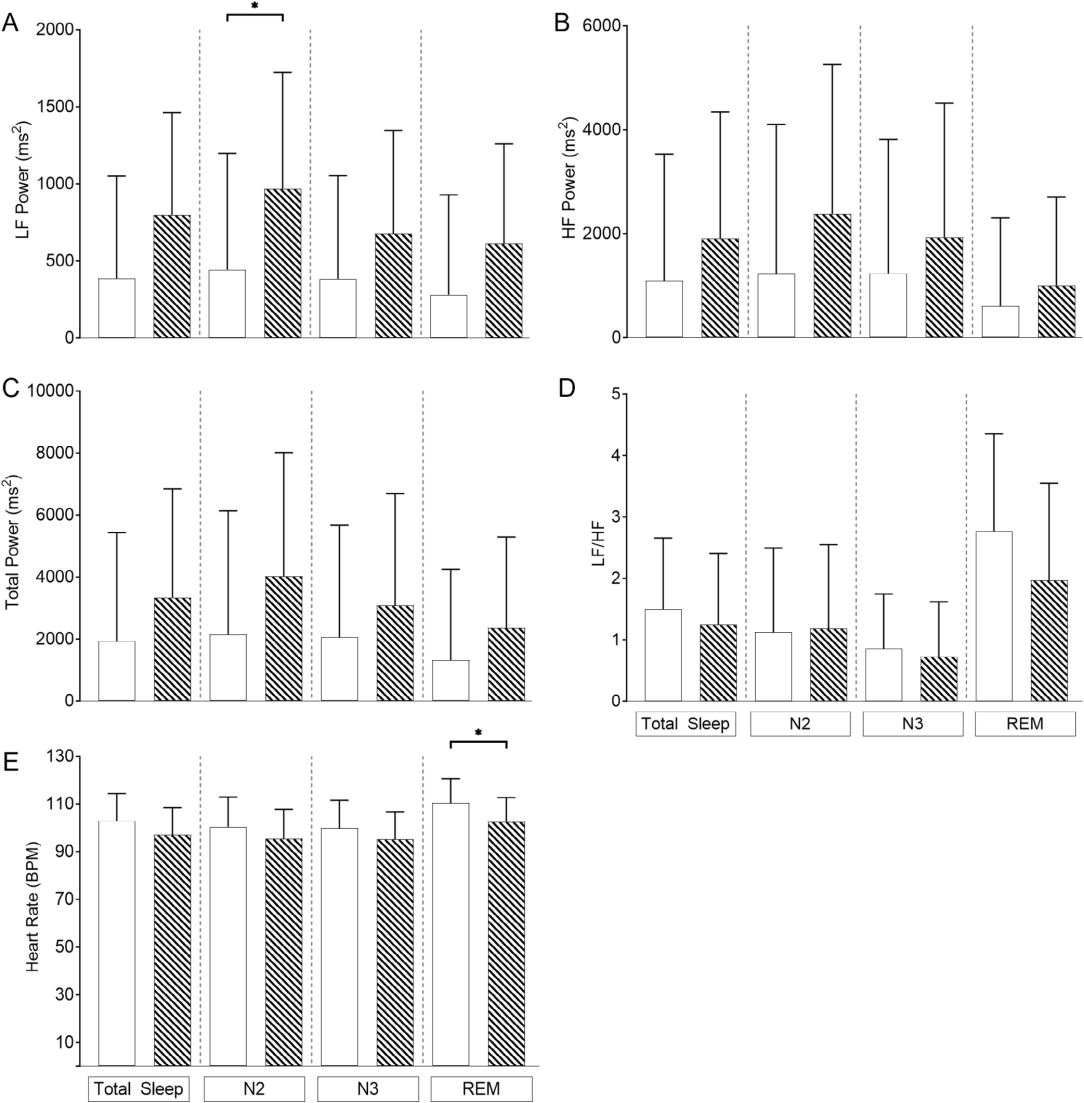
Differences in heart rate and frequency domain heart rate variability parameters using linear mixed‐effects modelling in children > 1 ≤ 6 years covarying for age, obstructive apnoea‐hypopnoea index (OAHI) and central apnoea‐hypopnoea index (CAHI) in children with Prader‐Willi syndrome (white bars) (*n* = 21) and typically developing (TD) children (hashed bars) (*n* = 21) for (A) LF power; (B) HF power; (C) total power; (D) sympathovagal balance (LF/HF) and (E) heart rate. Values are the adjusted mean and standard deviation. *p < 0.05.

**FIGURE 4:**
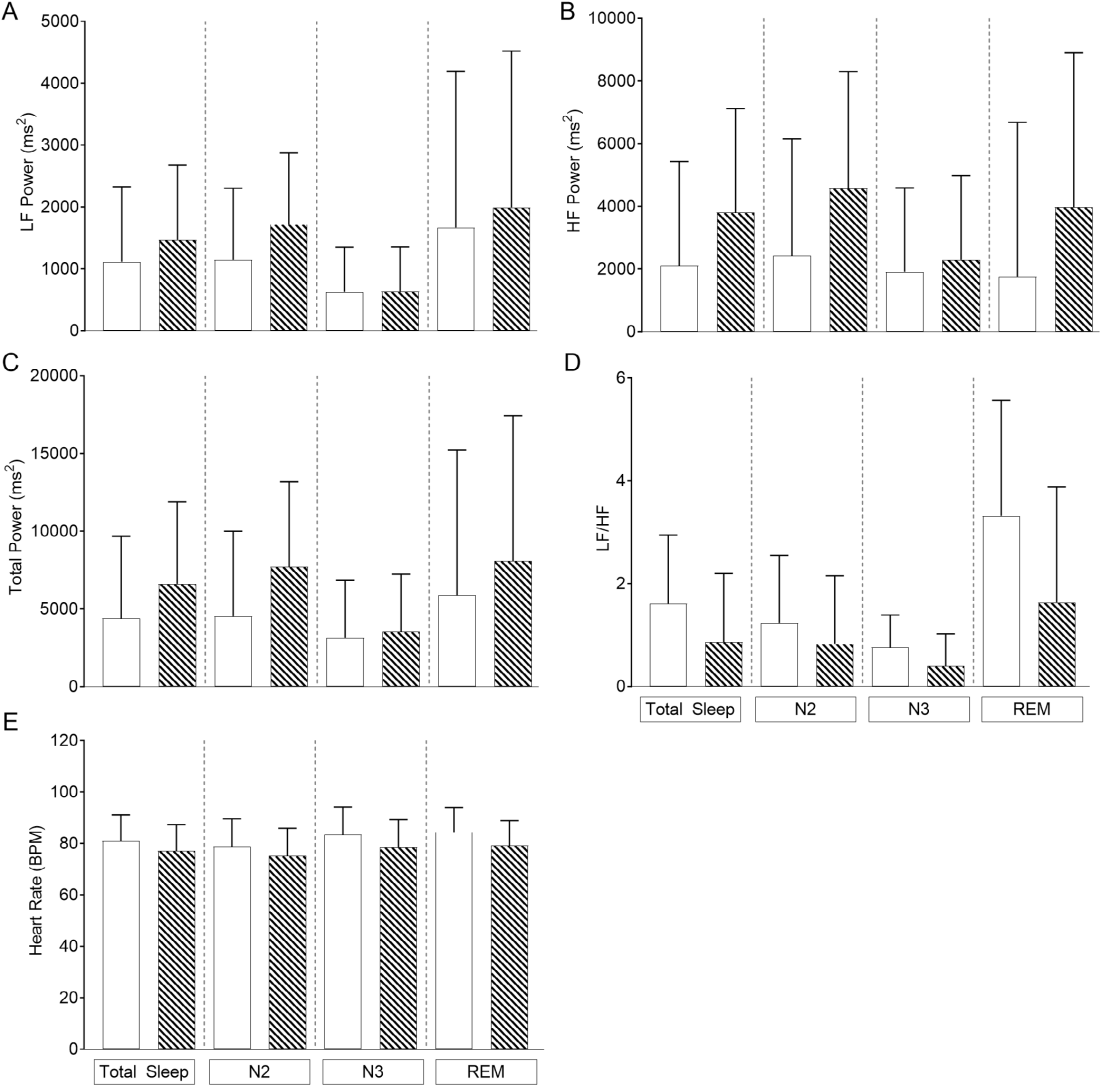
Differences in heart rate and frequency domain heart rate variability parameters using Linear mixed‐effects modelling in children > 6 years covarying for age, obstructive apnoea‐hypopnoea index (OAHI) and central apnoea‐hypopnoea index (CAHI) in children with Prader‐Willi syndrome (white bars) (*n* = 12) and typically developing (TD) children (hashed bars) (*n* = 12) for (A) LF power; (B) HF power; (C) total power; (D) sympathovagal balance (LF/HF) and (E) heart rate. Values are the adjusted mean and standard deviation.

As autonomic control matures rapidly during childhood, we repeated our mixed‐model analysis covarying for age, OAHI and CAHI in three age group categories: children ≤ 1 year (*n* = 17), > 1 ≤ 6 years (*n* = 21) and > 6 years (*n* = 12) of age. In the children ≤ 1 year of age (Figure 2), LF power was lower during total sleep, N2, N3 and REM sleep; total power was lower in N2 and REM sleep, and HR was higher in REM sleep in the children with PWS compared to controls. In the children > 1 ≤ 6 years of age (Figure 3) LF power was lower in N2, and HR was higher in REM sleep in the children with PWS compared to their controls. In the children > 6 years of age, there were no differences observed between groups (Figure 4).

Time‐domain mixed‐model analysis adjusting for age, OAHI and CAHI is presented in Table 2. In the group as a whole, children with PWS had significantly lower SDRR and pRR50 (*p* < 0.05 for both) in REM sleep when compared to controls. When children were stratified by age, children with PWS ≤ 1 year had significantly lower SDRR in REM sleep; however, no significant difference was found in children > 1 ≤ 6 and > 6 years.

**TABLE 2:** Time domain heart rate variability in sleep stages in children with Prader‐Willi syndrome (PWS) and typically developing (TD) children. Values and median (IQR).

	All children		≤ 1 year		> 1 ≤ 6 years		> 6 years	
	PWS	TD	PWS	TD	PWS	TD	PWS	TD
*N*	50	50	17	17	21	21	12	12
TS SDRR (ms)	30.9 (17.7, 50.8)	31.2 (24.0, 58.0)	17.9 (14.6, 27.6)	26.8 (23.2, 28.7)	34.4 (20.7, 51.8)	38.2 (25.6, 64.5)	48.6 (36.7, 89.0)	67.4 (39.8, 102.0)
TS RMSSD (ms)	30.3 (13.7, 54.5)	25.9 (19.4, 64.1)	16.0 (12.2, 28.3)	22.3 (19.0, 24.5)	32.2 (16.2, 59.2)	36.2 (17.1, 67.5)	54.1 (30.8, 92.6)	69.7 (33.7, 122.8)
TS pRR50 (%)	9.7 (0.8, 28.6)	8.2 (2.8, 28.4)	1.4 (0.2, 9.7)	3.5 (1.7, 5.8)	10.1 (1.1, 29.4)	15.2 (2.5, 29.6)	31.5 (10.3, 44.0)	37.4 (14.6, 60.8)
N2 SDRR (ms)	32.0 (16.2, 56.8)	31.5 (24.4, 69.2)	17.0 (15.2, 28.6)	25.2 (21.7, 29.2)	37.8 (19.1, 58.1)	41.5 (26.4, 73.1)	54.4 (36.3, 90.6)	74.2 (43.6, 107.2)
N2 RMSSD (ms)	32.8 (14.0, 62.1)	29.3 (20.2, 71.6)	14.2 (11.5, 29.1)	21.8 (19.0, 24.2)	37.1 (16.8, 68.6)	37.7 (16.7, 78.5)	56.0 (33.2, 99.0)	75.5 (36.6, 126.5)
N2 pRR50 (%)	10.7 (0.9, 33.2)	9.3 (2.5, 36.1)	1.1 (0.1, 8.5)	3.7 (1.7, 5.7)	13.0 (1.0, 32.2)	19.9 (2.2, 36.1)	35.0 (11.7, 56.0)	42.9 (16.3, 67.8)
N3 SDRR (ms)	30.9 (16.1, 54.6)	31.0 (22.0, 56.0)	16.2 (13.2, 29.2)	24.9 (21.1, 31.0)	33.8 (18.3, 62.2)	39.2 (22.2, 62.7)	33.4 (30.8, 77.4)	51.1 (33.4, 79.2)
N3 RMSSD (ms)	31.4 (18.2, 65.5)	31.5 (21.8, 63.0)	18.8 (13.7, 34.2)	24.5 (19.4, 31.5)	34.4 (19.6, 76.7)	42.6 (20.6, 72.5)	38.3 (28.8, 86.4)	50.6 (34.4, 93.2)
N3 pRR50 (%)	9.7 (1.4, 34.9)	10.3 (2.5, 29.5)	1.9 (0.3, 13.4)	4.2 (1.7, 10.3)	13.0 (1.9, 41.8)	18.6 (3.2, 32.6)	18.5 (8.2, 49.5)	29.4 (16.1, 56.4)
REM SDRR (ms)	26.0 (18.8, 36.5)*	32.7 (24.1, 55.2)	18.8 (15.9, 21.8)**	26.0 (23.0, 30.2)	26.0 (20.1, 29.5)	34.8 (25.0, 54.1)	56.1 (40.4, 97.1)	68.0 (41.1, 107.3)
REM RMSSD (ms)	17.2 (11.3, 23.5)	23.3 (14.3, 43.9)	12.0 (9.6, 15.1)	16.0 (13.0, 21.1)	17.8 (10.8, 21.9)	29.0 (14.0, 54.3)	40.1 (19.2, 65.0)	55.4 (28.3, 125.7)
REM pRR50 (%)	1.8 (0.4, 4.5)*	4.2 (1.0, 15.9)	0.5 (0.2, 0.9)	1.5 (0.7, 3.0)	2.3 (0.5, 4.1)	5.4 (1.2, 16.0)	14.3 (2.1, 27.6)	24.0 (7.0, 48.9)

*Note*: *p < 0.05 (in bold), **p < 0.01 (in bold); PWS vs. TD.

Abbreviations: pRR50, percentage of successive RR intervals differing by > 50 ms; REM, rapid eye movement; RMSSD, square root of the mean of the sum of the squares of differences between adjacent RR intervals; SDRR, standard deviation of R‐R intervals; TS, total sleep.

Linear mixed‐effects modelling results of the per cent change (%∆) in HR from wake to total sleep, N2, N3 and REM sleep are presented in Table 3. For the entire group, the change in HR from wake to REM was significantly less in children with PWS when compared to TD children. Children with PWS ≤ 1 year of age had a smaller change in HR from wake to N2, and children > 1 ≤ 6 years of age had a smaller fall in HR to REM. There were no significant differences between groups in falls in HR in children > 6 years of age.

**TABLE 3:** Percent change (%∆) in heart rate from wake before sleep onset to total sleep (TS), N2, N3 and REM sleep during the first cycle of sleep in children with Prader–Willi syndrome (PWS) and typically developing (TD) children. Values are median (IQR).

		All children (*n* = 50)		≤ 1 year (*n* = 17)		> 1 ≤ 6 years (*n* = 21)		> 6 years (*n* = 12)	
		PWS	TD	PWS	TD	PWS	TD	PWS	TD
	*n*	36	32	9	6	16	14	11	12
%∆ N2		−5.4 (−11.0–1.6)	−7.8 (−12.3, −2.0)	−9.2 (−12.6, −3.2)*	−13.4 (−17.4, −11.5)	−5.7 (−10.8, −4.1)	−6.5 (−12.9, −3.3)	−0.7 (−9.5, 6.2)	−2.0 (−8.8, 0.9)
	*n*	36	35	10	7	15	16	11	12
%∆ N3		−13.0 (−17.2, −5.9)	−12.1 (−16.8, −5.7)	−16.0 (−19.3, −12.3)	−16.8 (−20.5, −15.4)	−13.6 (−17.3, −6.8)	−13.6 (−19.4, −8.9)	−5.8 (−13.0, 0.4)	−3.8 (−10.4, 6.1)
	*n*	38	34	11	7	16	16	11	11
%∆ REM		−4.6 (−9.1, 1.5)*	−10.2 (−14.1, −4.5)	−8.6 (−12.9, −3.7)	−13.6 (−16.0, −5.9)	−2.5 (−9.6, 2.0)**	−10.1 (−13.8, −6.1)	−1.6 (−8.9, 4.2)	−5.6 (−13.6, 6.5)
	*n*	38	35	11	7	16	16	11	12
%∆ TS		−7.9 (−13.7, −3.0)	−10.4 (−15.2, −6.2)	−13.7 (−16.1, −7.6)	−15.1 (−16.9, −11.5)	−7.6 (−11.8, −4.0)	−11.1 (−15.5, −7.4)	−3.2 (−8.6, −0.1)	−6.2 (−10.0, 3.2)

*Note*: *p < 0.05 (in bold), **p < 0.01 (in bold); PWS vs. TD.

Abbreviation: REM, rapid eye movement.

When classified according to their dipping profile, there were no significant differences between children with PWS and TD children in the numbers of children in each dipping profile. In the non‐dipping group, there were 19 children (50%) with PWS and 13 (37%) controls; 13 (34%) children with PWS and 18 (51%) controls were dippers; 3 (8%) children with PWS and 2 (6%) controls were extreme dippers; and 3 (8%) children with PWS and 2 (6%) controls were reverse dippers.

## 4 Discussion

This is the first study to compare autonomic control in children with PWS naïve to GH treatment to that of TD children while controlling for age and severity of OSA and CSA. In the group as a whole, when analysed in the frequency domain, we found lower LF power in N2 sleep in the children with PWS. When stratified by age, this reduced LF was more marked in the children ≤ 1 year of age, occurring in all sleep states, and in addition, total power was reduced in N2 and REM sleep. No differences were found in the older children. Similarly, time domain analysis of HRV found lower SDRR, also reflecting reduced Total power consequent on reduced parasympathetic and sympathetic activity in the younger children. In support of our findings suggesting reduced autonomic control of HR, the nocturnal fall in HR was also reduced. Together, these findings suggest that autonomic control is dampened in young children with PWS; however, this appears to improve with age, as no differences were identified in children over 6 years of age.

Considering age is important when examining autonomic control, dividing our cohort into age groups accounted for the rapid development in autonomic control from birth to middle and late childhood and is a strength of our study (Harteveld et al. 2021). This was the first study to investigate HRV in children under 1 year of age, with the study by Brito et al., including children with PWS aged 10 ± 3 years (Brito et al. 2021), the study by Richer et al., including children with PWS aged 11 ± 4 years (Richer et al. 2023) and the study by Debs et al., including children with PWS between 1.1 and 17.1 years (Debs et al. 2024). Over the first 6 months of life, there is an increase in both LF and HF power (Yiallourou et al. 2013), and this continues to around the age of 5 years (Harteveld et al. 2021). Our finding that no differences were identified in older children above 6 years of age suggests that this impaired autonomic control may be due to delayed maturation of cardiovascular control in children with PWS compared to TD children.

Differences in our study findings and those of previous studies of HRV in children with PWS are likely attributable to study design and methods. Brito et al. conducted HRV analysis on the first two consecutive 5‐min uninterrupted periods of N2, N3 and REM sleep, without significant apneas/hypopneas or arousals (Brito et al. 2021). Similar to our study in children over 6 years, they showed no difference in the frequency domain HRV; however, in the time domain, they found that children with PWS had significantly lower total HRV and parasympathetic modulation during N3 sleep when compared to both obese and lean controls (Brito et al. 2021). Debs et al. also investigated HRV in 5‐min ECG periods without respiratory or arousal events and found similar findings to ours in both time and frequency domain analyses, where children with PWS had significantly lower LF power in the frequency domain analysis and Total power (SDRR) in the time domain when compared to TD children (Debs et al. 2024). The methodology of Brito et al. of performing frequency domain analyses only in the first two consecutive 5‐min periods of stable sleep differs from our methodology and could potentially explain contrasting findings to the current study, where we analysed HRV in every 2‐min period that fulfilled our inclusion criteria over the entire night (Brito et al. 2021). In addition, both Brito et al. and Debs et al. investigated HRV only during periods of stable sleep not interrupted by respiratory events, whereas our analysis included both central and obstructive respiratory events, with children being matched for OSA and CSA severity, and our analysis co‐varied for this to mitigate the effects of OSA and CSA on HRV. We have previously shown that HRV in TD children with OSA is dampened, regardless of whether respiratory events were included or excluded from the analyses (Nisbet et al. 2013), highlighting the importance of controlling for SDB when investigating the effect of PWS on autonomic control. The study by Richer et al. investigated children with PWS during wakefulness compared to controls matched for age and BMI. They analysed HRV in 5 min of supine and 5 min after a 70° head‐up tilt (Richer et al. 2023). They showed no difference in either time‐ or frequency‐domain HRV during the supine period. These findings are consistent with our study in our older children. Their study, however, identified reduced changes in HR during both the head‐up tilt and Valsalva manoeuvre, and increased sympathovagal balance (LF/HF) during the head‐up tilt (Richer et al. 2023). This suggests an autonomic response to a challenge may persist in older children, with a blunted sympathetically mediated HR response to the head‐up tilt or higher vagally mediated parasympathetic tone in children with PWS. A major difference to the current study is that the study by Richer et al. was conducted during wakefulness rather than sleep, and it is unknown whether children in their analysis had OSA and CSA.

Our study also identified that the younger children with PWS had elevated HR in REM sleep with no differences identified in older children. Consistent with our study, Richer et al. found no differences in HR between children with PWS and TD children when awake (Richer et al. 2023). In contrast to our study, Brito et al. found that children with PWS had significantly lower HR when compared to both obese and lean controls in N2 and N3 sleep (Brito et al. 2021) and Debs et al. found reduced HR changes with deep breathing and active standing in 22% and 47% of the children with PWS, respectively (Debs et al. 2024). The increased HR in REM sleep observed in the younger children in our study may be due to a maturational delay, as HR decreases with increasing postnatal age (Harteveld et al. 2021).

Our study was the first to match controls for OSA and CSA severity as it has been found that TD children with OSA have elevated and dysregulated sympathetic activity when assessed using HRV and urinary catecholamines (a measure of overall sympathetic activity in the body), which may contribute to cardiovascular consequences (O'Driscoll et al. 2011; Nisbet et al. 2014). In our study, only one child with PWS had severe OSA (OAHI ≥ 10 events/h) and 2 had moderate OSA, with the majority (84%) having primary snoring, whereas the children in the study by Brito et al. had a mean apnoea‐hypopnoea index of 13.9 ± 27.0 and 46% of children with PWS had OSA in the study by Debs et al. indicating that the children in their study had more severe OSA. It is not reported if children in the study by Richer et al. had either OSA or CSA (Richer et al. 2023). Despite differences in methodology, our findings support these previous studies and show that cardiovascular control is reduced in children with PWS compared to matched TD children, even when accounting for OSA/CSA severity, and importantly this is most marked in children under 6 years of age.

The previous studies investigated children with PWS who were on GH (Debs et al. 2024) or had a number of the children on GH (Brito et al. 2021; Richer et al. 2023), which is known to impact autonomic function (Leong et al. 2001). For example, it has been reported that adults with GH deficiency during wakefulness have significantly reduced LF power and sympathovagal balance and increased HF power when compared to controls (Leong et al. 2000, 2001), which significantly improved after GH replacement therapy (Leong et al. 2001). As such, we only included GH naïve children with PWS to eliminate the potential impact of GH on autonomic control.

This is the first study to investigate nocturnal dipping in children with PWS. In the whole group, we found that children with PWS had reduced nocturnal dipping of HR from wake to REM sleep compared to TD children. Similarly, children with PWS > 1 ≤ 6 years had reduced dipping to REM sleep, and children with PWS ≤ 1 had reduced dipping to N2 sleep. We did not identify any differences between groups in the proportion of children in each dipping profile. The reduced dipping of HR during sleep further indicates dysfunction in autonomic control in children with PWS, which is prominent in younger children under 6 years.

The strengths of our study are the large sample size given the rarity of PWS, and that we tightly controlled for age, OSA, CSA and BMI. However, the limitations of the current study are the low sample sizes after age stratification, which was particularly prominent in children > 6 years of age (*n* = 12), as well as the fact that we did not adjust for multiple comparisons. Notwithstanding, our cross‐sectional data suggest that autonomic dysfunction is worst in children with PWS ≤ 1 year of age and is less significant > 6 years. It will be important to conduct longitudinal studies to confirm this apparent improvement in autonomic function after 6 years of age. The well‐described increased cardiovascular risk in adulthood (Van Vliet et al. 2004; Tauber et al. 2008) is not consistent with our findings of improvement in autonomic function with age throughout childhood, suggesting that either autonomic dysfunction in sleep is not linked to adverse cardiovascular outcomes, or dysfunction appears again at older ages. Detailed evaluation of this aspect of cardiovascular functioning, and the impact of treatments such as GH, which has been shown to improve autonomic control in other patient groups (Leong et al. 2000, 2001), has the potential to improve understanding of this potential influence on long‐term cardiovascular health in people with PWS.

## 5 Conclusions

Our study highlights that children under 6 years of age with PWS present cardiac autonomic imbalance due to reduced sympathetic and parasympathetic activity during sleep, and have significantly elevated HR and reduced nocturnal dipping from wake to REM sleep compared to matched controls. Further investigation of autonomic function in children with PWS is required to further understand the relationship of autonomic dysfunction to cardiovascular risk in this vulnerable population, and also the impacts of treatments such as growth hormone.

## Author Contributions

**Okkes R. Patoglu:** investigation, formal analysis, writing – original draft. **Lisa M. Walter:** formal analysis, writing – review and editing. **Georgina Plunkett:** formal analysis, writing – review and editing. **Margot J. Davey:** writing – review and editing. **Gillian M. Nixon:** funding acquisition, writing – review and editing, supervision. **Bradley A. Edwards:** writing – review and editing, supervision. **Rosemary S. C. Horne:** conceptualization, funding acquisition, writing – review and editing, supervision, project administration.

## Ethics Statement

Ethical approval for this study was granted by Monash Health (RES22‐0000035L) and Monash University Human Research Ethics Committees.

## Consent

Parents provide written informed consent for their child's data to be used for research.

## Conflicts of Interest

The authors declare no conflicts of interest.

## Data Availability

The data that support the findings of this study are available from RSC Horne, but restrictions apply to the availability of these data, which were used under licence for the current study and so are not publicly available. Data are however available from the authors upon reasonable request and with permission of RSC Horne.

## References

[jsr70094-bib-0001] Abel, F. , H. L. Tan , V. Negro , et al. 2019. “Hypoventilation Disproportionate to OSAS Severity in Children With Prader‐Willi Syndrome.” Archives of Disease in Childhood 104: 166–171. 10.1136/archdischild-2017-314282 30007944

[jsr70094-bib-0002] Andriessen, P. , A. M. Koolen , R. C. Berendsen , et al. 2003. “Cardiovascular Fluctuations and Transfer Function Analysis in Stable Preterm Infants.” Pediatric Research 53, no. 1: 89–97. 10.1203/00006450-200301000-00016. 12508086

[jsr70094-bib-0003] Ben‐Dov, I. Z. , J. D. Kark , D. Ben‐Ishay , J. Mekler , L. Ben‐Arie , and M. Bursztyn . 2007. “Blunted Heart Rate Dip During Sleep and All‐Cause Mortality.” Archives of Internal Medicine 167: 2116–2121. 17954807 10.1001/archinte.167.19.2116

[jsr70094-bib-0004] Berry, R. B. , R. Brooks , C. Gamaldo , et al. 2017. “AASM Scoring Manual Updates for 2017 (Version 2.4).” Journal of Clinical Sleep Medicine 13: 665–666. 28416048 10.5664/jcsm.6576 PMC5406946

[jsr70094-bib-0005] Berry, R. B. , R. Budhiraja , D. J. Gottlieb , et al. 2012. “Rules for Scoring Respiratory Events in Sleep: Update of the 2007 AASM Manual for the Scoring of Sleep and Associated Events. Deliberations of the Sleep Apnea Definitions Task Force of the American Academy of Sleep Medicine.” Journal of Clinical Sleep Medicine 8: 597–619. 23066376 10.5664/jcsm.2172 PMC3459210

[jsr70094-bib-0006] Berry, R. B. , S. F. Quan , and A. Abreu . 2020. The AASM Manual for the Scoring of Sleep and Associated Events: Rules, Terminology and Technical Specifications. American Academy of Sleep Medicine.

[jsr70094-bib-0007] Brito, L. C. , T. Queiroga , R. R. Franco , et al. 2021. “Cardiac Autonomic Control During Non‐REM and REM Sleep Stages in Paediatric Patients With Prader‐Willi Syndrome.” Journal of Sleep Research 30: e13165. 32812310 10.1111/jsr.13165

[jsr70094-bib-0008] Butler, M. G. , A. M. Manzardo , J. Heinemann , C. Loker , and J. Loker . 2017. “Causes of Death in Prader‐Willi Syndrome: Prader‐Willi Syndrome Association (USA) 40‐Year Mortality Survey.” Genetics in Medicine 19: 635–642. 27854358 10.1038/gim.2016.178 PMC5435554

[jsr70094-bib-0009] Butler, M. G. , A. K. Victor , and L. T. Reiter . 2023. “Autonomic Nervous System Dysfunction in Prader‐Willi Syndrome.” Clinical Autonomic Research 33: 281–286. 36515769 10.1007/s10286-022-00909-7

[jsr70094-bib-0010] Cassidy, S. B. , S. Schwartz , J. L. Miller , and D. J. Driscoll . 2012. “Prader‐Willi Syndrome.” Genetics in Medicine 14: 10–26. 22237428 10.1038/gim.0b013e31822bead0

[jsr70094-bib-0011] Debs, R. , G. Diene , J. Cortadellas , et al. 2024. “Cardiovascular Autonomic Dysfunction and Sleep Abnormalities in Children With Prader‐Willi Syndrome.” Clinical Autonomic Research 35: 243–255. 39633031 10.1007/s10286-024-01083-8

[jsr70094-bib-0012] DiMario, F. J. , B. Dunham , J. A. Burleson , J. Moskovitz , and S. B. Cassidy . 1994. “An Evaluation of Autonomic Nervous System Function in Patients With Prader‐Willi Syndrome.” Pediatrics 93, no. 1: 76–81. 10.1542/peds.93.1.76. 8265328

[jsr70094-bib-0013] Fagard, R. H. 2009. “Dipping Pattern of Nocturnal Blood Pressure in Patients With Hypertension.” Expert Review of Cardiovascular Therapy 7: 599–605. 19505275 10.1586/erc.09.35

[jsr70094-bib-0014] Felix, O. , A. Amaddeo , J. Olmo Arroyo , et al. 2016. “Central Sleep Apnea in Children: Experience at a Single Center.” Sleep Medicine 25: 24–28. 27823711 10.1016/j.sleep.2016.07.016

[jsr70094-bib-0015] Francis, J. , G. Plunkett , M. Shetty , et al. 2024. “Autonomic Cardiovascular Control Is Unaffected in Children Referred for Assessment of Excessive Daytime Sleepiness.” Journal of Sleep Research 34: e14318. 39147593 10.1111/jsr.14318 PMC11744242

[jsr70094-bib-0016] Gillett, E. S. , and I. A. Perez . 2016. “Disorders of Sleep and Ventilatory Control in Prader–Willi Syndrome.” Diseases 4: 23. 28933403 10.3390/diseases4030023 PMC5456282

[jsr70094-bib-0017] Harteveld, L. M. , I. Nederend , A. D. J. Ten Harkel , et al. 2021. “Maturation of the Cardiac Autonomic Nervous System Activity in Children and Adolescents.” Journal of the American Heart Association 10: e017405. 33525889 10.1161/JAHA.120.017405 PMC7955328

[jsr70094-bib-0018] Hedgeman, E. , S. P. Ulrichsen , S. Carter , et al. 2017. “Long‐Term Health Outcomes in Patients With Prader‐Willi Syndrome: A Nationwide Cohort Study in Denmark.” International Journal of Obesity 41: 1531–1538. 28634363 10.1038/ijo.2017.139

[jsr70094-bib-0019] Iber, C. , S. Ancoli‐Israel , A. L. Chesson , and S. F. Quan . 2007. The AASM Manual for the Scoring of Sleep and Associated Events. American Academy of Sleep Medicine.

[jsr70094-bib-0020] Kritzinger, F. E. , S. Al‐Saleh , and I. Narang . 2011. “Descriptive Analysis of Central Sleep Apnea in Childhood at a Single Center.” Pediatric Pulmonology 46: 1023–1030. 21520440 10.1002/ppul.21469

[jsr70094-bib-0021] Leong, K. S. , P. Mann , M. Wallymahmed , I. A. MacFarlane , and J. P. Wilding . 2000. “Abnormal Heart Rate Variability in Adults With Growth Hormone Deficiency.” Journal of Clinical Endocrinology and Metabolism 85, no. 2: 628–633. 10.1210/jcem.85.2.6396. 10690867

[jsr70094-bib-0022] Leong, K. S. , P. Mann , M. Wallymahmed , I. A. MacFarlane , and J. P. Wilding . 2001. “The Influence of Growth Hormone Replacement on Heart Rate Variability in Adults With Growth Hormone Deficiency.” Clinical Endocrinology 54, no. 6: 819–826. 10.1046/j.1365-2265.2001.01267.x. 11422118

[jsr70094-bib-0023] Lu, A. , F. Luo , C. Sun , X. Zhang , L. Wang , and W. Lu . 2020. “Sleep‐Disordered Breathing and Genetic Findings in Children With Prader‐Willi Syndrome in China.” Annals of Translational Medicine 8: 989. 32953789 10.21037/atm-20-4475 PMC7475489

[jsr70094-bib-0024] Malik, M. J. , A. Thomas Bigger , J. Camm , et al. 1996. “Heart Rate Variability: Standards of Measurement, Physiological Interpretation, and Clinical Use. Task Force of the European Society of Cardiology and the North American Society of Pacing and Electrophysiology.” European Heart Journal 17: 354–381. 8737210

[jsr70094-bib-0025] Marcus, C. L. , L. J. Brooks , K. A. Draper , et al. 2012b. “Diagnosis and Management of Childhood Obstructive Sleep Apnea Syndrome.” Pediatrics 130: e714–e755. 22926176 10.1542/peds.2012-1672

[jsr70094-bib-0026] Marcus, K. A. v. d. , J. A. van Alfen‐ Velden , B. J. Otten , et al. 2012a. “Cardiac Evaluation in Children With Prader‐Willi Syndrome.” Acta Paediatrica 101, no. 5: e225‐31. 10.1111/j.1651-2227.2011.02570.x. 22181352

[jsr70094-bib-0027] Nisbet, L. C. , S. R. Yiallourou , G. M. Nixon , et al. 2013. “Nocturnal Autonomic Function in Preschool Children With Sleep‐Disordered Breathing.” Sleep Medicine 14: 1310–1316. 24091143 10.1016/j.sleep.2013.07.010

[jsr70094-bib-0028] Nisbet, L. C. , S. R. Yiallourou , L. M. Walter , and R. S. Horne . 2014. “Blood Pressure Regulation, Autonomic Control and Sleep Disordered Breathing in Children.” Sleep Medicine Reviews 18: 179–189. 23850404 10.1016/j.smrv.2013.04.006

[jsr70094-bib-0029] O'Driscoll, D. M. , R. S. Horne , M. J. Davey , et al. 2011. “Increased Sympathetic Activity in Children With Obstructive Sleep Apnea: Cardiovascular Implications.” Sleep Medicine 12, no. 5: 483–488. 10.1016/j.sleep.2010.09.015. 21521626

[jsr70094-bib-0030] Richer, L. P. , Q. Tan , M. G. Butler , et al. 2023. “Evaluation of Autonomic Nervous System Dysfunction in Childhood Obesity and Prader‐Willi Syndrome.” International Journal of Molecular Sciences 24, no. 9: 8013. 10.3390/ijms24098013. 37175718 PMC10179129

[jsr70094-bib-0031] Schluter, B. , D. Buschatz , E. Trowitzsch , F. Aksu , and W. Andler . 1997. “Respiratory Control in Children With Prader‐Willi Syndrome.” European Journal of Pediatrics 156: 65–68. 9007495 10.1007/s004310050555

[jsr70094-bib-0032] Shypailo, R. 2020. Age‐Based Pediatric Growth Reference Charts Baylor College of Medicine, Children's Nutrition Research Center, Body Composition Laboratory.

[jsr70094-bib-0033] Snyder, F. , J. A. Hobson , D. F. Morrison , and F. Goldfrank . 1964. “Changes in Respiration, Heart Rate, and Systolic Blood Pressure in Human Sleep.” Journal of Applied Physiology 19: 417–422. 14174589 10.1152/jappl.1964.19.3.417

[jsr70094-bib-0034] Tan, H. L. , and D. S. Urquhart . 2017. “Respiratory Complications in Children With Prader Willi Syndrome.” Paediatric Respiratory Reviews 22: 52–59. 27839656 10.1016/j.prrv.2016.08.002

[jsr70094-bib-0035] Tauber, M. , G. Diene , C. Molinas , and M. Hebert . 2008. “Review of 64 Cases of Death in Children With Prader‐Willi Syndrome (PWS).” American Journal of Medical Genetics. Part A 146A: 881–887. 18324685 10.1002/ajmg.a.32131

[jsr70094-bib-0036] Van Vliet, G. , C. L. Deal , P. A. Crock , Y. Robitaille , and L. L. Oligny . 2004. “Sudden Death in Growth Hormone‐Treated Children With Prader‐Willi Syndrome.” Journal of Pediatrics 144, no. 1: 129–131. 10.1016/j.jpeds.2003.09.049. 14722532

[jsr70094-bib-0037] Walter, L. M. , G. M. Nixon , M. J. Davey , V. Anderson , A. M. Walker , and R. S. Horne . 2013. “Autonomic Dysfunction in Children With Sleep Disordered Breathing.” Sleep and Breathing 17, no. 2: 605–613. 10.1007/s11325-012-0727-x. 22684854

[jsr70094-bib-0038] Yiallourou, S. R. , N. B. Witcombe , S. A. Sands , A. M. Walker , and R. S. Horne . 2013. “The Development of Autonomic Cardiovascular Control Is Altered by Preterm Birth.” Early Human Development 89, no. 3: 145–152. 10.1016/j.earlhumdev.2012.09.009. 23058299

